# Effect of Nourishing “Yin” Removing “Fire” Chinese Herbal Mixture on Hypothalamic Mammalian Target of Rapamycin Expression during Onset of Puberty in Female Rats

**DOI:** 10.1155/2015/157846

**Published:** 2015-09-17

**Authors:** Gulan Zeng, Xinghui Han, Jian Yu, Yonghong Wang, Zhanzhuang Tian

**Affiliations:** ^1^Department of Integrative Medicine, Children's Hospital of Fudan University, 399 Wanyuan Road, Shanghai 201102, China; ^2^Department of Neurobiology and Integrative Medicine, Shanghai Medical College of Fudan University, 130 Dong An Road, Shanghai 200032, China

## Abstract

*Aim*. The present study aims to investigate the effects of nourishing “Yin” removing “Fire” (NYRF) Chinese herbal mixture on puberty onset and hypothalamic mTOR expression in female rats. *Materials and Methods*. Forty female 20-day-old Sprague-Dawley rats were randomly divided into Chinese herbal mixture (CHM) and normal saline (NS) groups. Rats in CHM and NS were treated with NYRF mixture and normal saline, respectively, from d22. Rats in each group were sacrificed on d28, d31, and d34. Serum luteinizing hormone (LH), follicle stimulating hormone (FSH), and estradiol (E2) levels were analyzed by ELISA. Hypothalamic mTOR mRNA expression levels were determined by RT-PCR and hypothalamic p-mTOR protein levels were assayed by western blot. *Results*. The vaginal opening time in CHM group was significantly delayed (*P* < 0.05). On d31, in comparison with NS group, the coefficients of uteri and ovaries, levels of serum LH and E2, and the expression levels of hypothalamic mTOR mRNA and p-mTOR protein were significantly lower in CHM group (*P* < 0.05). *Conclusion*. The mechanism by which the nourishing “Yin” removing “Fire” Chinese herbal mixture delays puberty onset may be associated with the inhibition of the hypothalamic mTOR signaling.

## 1. Introduction

Adolescence is a transitional phase from childhood to adulthood when tissues and organs in the whole body are gradually developed. The most significant feature of adolescence is rapid maturation of the reproductive system, whereby the individuals attain reproductive capacity [1]. Puberty onset is initiated by the activation of hypothalamus-pituitary-gonadal axis (HPGA) [2], in which the key step is excitatory activation of gonadotropin releasing hormone (GnRH) pulse generator and increased secretion of GnRH [3] that stimulates the secretion of pituitary gonadotropins (LH and FSH). The gonadotropins can then stimulate the peripheral gonads to attain maturity and secrete gonadal hormones and form sperms or eggs.

The onset of puberty is a multifactorial and multilevel process involving many factors of the complex neuroendocrine regulatory networks. The mammalian target of rapamycin (mTOR) is an atypical serine-threonine protein kinase that belongs to the phosphatidylinositol kinase-related kinase (PIKK) family [4, 5]. mTOR, an evolutionarily conserved Ser/Thr protein kinase, is an important regulatory protein. It functions as an extracellular nutrient and as energy and growth factor sensor and regulates multiple signaling pathways. It plays a central role in cell growth, proliferation, differentiation, and apoptosis [6, 7]. In 2009, Roa et al. [8] first reported the involvement of hypothalamic mTOR signaling in the control of puberty onset. Their study found that, in pubertal female rats, LH secretion was significantly increased by intracerebroventricular (i.c.v.) injection of l-leucine (an agonist of mTOR signaling). Conversely, blockade of central mTOR signaling by rapamycin caused significant decrease of LH and E2 levels, late vaginal opening, and ovarian and uterine atrophy. This shows that the hypothalamic mTOR signaling plays an important role in the onset of puberty, and inhibition of the hypothalamic mTOR signaling may delay puberty onset.

Early puberty is a variation of normal development of puberty, and exaggerated early puberty can take the form of precocious puberty, characterized by early sexual development with early increase in breast or testicle development, appearance of pubic hair, early age of menarche or first nocturnal emission, and so forth. Early appearance of these series of physiological changes can cause abnormal psychological behavior in children, compromising their final adult height, and may increase the risk of adult cardiovascular and metabolic diseases such as hypertension, obesity, and diabetes [9–13]. Chinese herbal mixtures have been clinically employed for early puberty or precocious puberty treatment several decades ago. These herbal mixtures can delay the onset of puberty, gradually counteract the early development of secondary sexual characteristics, retract the ovary and uterus volume, slow down the growth rate of bones, and reduce the height damage caused by precocious puberty [14–16].

The mechanism by which the nourishing “Yin” removing “Fire” (NYRF) herbal mixture delays puberty onset may be a multitarget and multilevel regulation process. Our previous animal studies [16–19] showed that this mixture can suppress the expression of hypothalamic Kiss-1/kisspeptin and NKB/NK3R, reduce the release of hypothalamic excitatory amino acid neurotransmitters, increase the release of inhibitory amino acid neurotransmitters, and display an inhibitory effect on GnRH neurons, thereby delaying the onset of puberty. In the current study, the hypothalamic mTOR signaling is studied to explore the effects of the NYRF mixture on the expression of hypothalamic mTOR mRNA and p-mTOR protein, and we have further elucidated the possible mechanism by which this Chinese herbal mixture delays puberty onset.

## 2. Materials and Methods

### 2.1. Ethic Statement

The study was approved by the Ethic Committee of Pediatric Research Ethics Board of Clinical Pharmacology Base, Fudan University (number [2012]034, date of approval by ethic committee: 2012/03/07).

### 2.2. Preparation of Herbal Mixture

The Chinese herbal mixture is an original prescription from our Department of Integrative Medicine, Children's Hospital of Fudan University (Shanghai pharmacists system number Z05170908). The mixture mainly consists of* Rehmannia glutinosa* (Sheng-Di-Huang),* Scrophularia buergeriana* (Xuan-Shen),* Anemarrhena asphodeloides* (Zhi-Mu),* Cortex Phellodendri* (Huang-Bai), and so forth (Table 1). The mixture was prepared by traditional water extraction-alcohol precipitation method using a thermostat electric set (Zhengzhou Great Wall Scientific Industrial & Trade Co. Ltd.) to decoct the above-mentioned crude drugs for 40 min. Then the thermostat electric set was refilled with water for decocting for another 40 min. The extracted liquid was then collected and concentrated by a rotary evaporator (Buchi, Switzerland). Following this, absolute ethanol was slowly added to dilute the mixture till a final concentration of 60%, and then the mixture was incubated at 4°C for 72 h. Finally, ethanol was removed by the rotary evaporator and the crude drug was obtained at a final concentration of 2.7 g per mL.

### 2.3. Animals

Forty 20-day-old female Sprague-Dawley rats were purchased from Shanghai SLAC Laboratory Animal Co. (Shanghai, China) (license number: SCXK (Shanghai) 2012-0002). Animals were housed in Department of Neurobiology and Integrative Medicine of Fudan University. Animals had free access to food and water with controlled ambient temperature (24°C ± 2°C) and humidity (67%  ±  1.5%) with a 12/12 (light/dark) schedule in a room shielded from outside noise.

### 2.4. Experimental Design

Forty female SD rats were randomly divided into Chinese herbal mixture (CHM) and normal saline (NS) groups (*n* = 20 each). Starting from d22, rats in the CHM and NS groups were continuously gavaged every morning (8:00) and evening (18:00) with the Chinese herbal mixture or an equal volume of saline by a lavage needle, respectively, at the dose of 1 mL/100 g body weight (equivalent to the smallest dose used in clinic for the treatment of precocious puberty), until sacrificed. Rats were fasted the night before sacrificing from 20:00 till the next morning and were sacrificed 1 h after the last gavage on the morning of d28, d31, or d34, respectively. From d28, rats were observed for the day of vaginal opening (VO). Five rats in each group were sacrificed before puberty onset at d28 and d31 and ten rats in each group were sacrificed at d34 (all rats in NS showed VO by this time). The rats were weighed and anesthetized by intraperitoneal injection of 10% chloral hydrate (0.4 mL/100 g) before being sacrificed. The uteri and ovaries were immediately dissected out of the surrounding fat by opening the abdominal cavity and weighed to evaluate the organ coefficients according to the organ index formula ([organ wet weight (g)/body weight (g)] × 10^−4^). Subsequently, ovary samples were fixed with 4% paraformaldehyde solution, embedded with paraffin, sectioned, and stained with hematoxylin and eosin (HE).

### 2.5. Hormone Level Detection

Blood samples were derived from the jugular vein of rats anesthetized by intraperitoneal injection of 10% choral hydrate (0.4 mL/100 g) one hour after the last gavage on the morning of the same day. The serum was then separated using a high-speed freezing centrifuge (Heraeus, Germany) and stored at −80°C until assayed. Serum LH, FSH, and E2 levels were determined using ELISA Kits (eBioscience, USA) according to the manufacturer's specifications. The sensitivity of the kit for E2 was 1.7 pg/mL; the intra-assay coefficient was 4.5%. For sensitivity of the kit for LH, the assay sensitivity was 0.3 mIU/mL, and the intra-assay coefficient was 2.6%. For sensitivity of the kit for FSH, the assay sensitivity was 0.28 mIU/mL, and the intra-assay coefficient was 6%.

### 2.6. Real-Time Reverse Transcriptase-PCR (RT-PCR) Analysis

The effects of the Chinese herbal mixture on mTOR mRNA expression in the hypothalami of rats were detected by RT-PCR, performed in triplicate. Total RNA was isolated using the Direct-zol RNA MiniPrep Kit (Zymo Research Corp., USA) and RNA was reverse transcribed into cDNA using the 5x All-in-One RT MasterMix (ABM, Canada) (20 *μ*L volume) according to the manufacturer's supplied protocols. KAPA SYBR rapid quantitative PCR MasterMix (2x) (KAPA Biosystems Inc., USA) was added to 20 *μ*L of the reaction solution for RT-PCR. The amplification procedure conditions were as follows: predenaturation at 95°C for 3 min followed by denaturation at 95°C for 5 s, then annealing at 60°C, and extension for 30 s with a total of 40 amplification cycles. GAPDH was used as internal standard, using the 2^−ΔΔCt^ method to calculate the relative expression levels. The primers were synthesized by Shanghai Sangon Biotech Inc. (Shanghai, China) and are shown in Table 2.

### 2.7. Western Blot Analysis

Three rats in each group were randomly selected for hypothalamic p-mTOR expression analysis. Hypothalamus samples were lysed in RIPA buffer (Beyotime Institute of Biotechnology, China) and the amount of total cellular protein was determined by BCA protein assay kit (Thermo Scientific). Then, 5x sample loading buffer (Beyotime Institute of Biotechnology, China) was added at the ratio of 1 : 4 and incubated in the boiling water bath for 5 min, and 40 *μ*g of samples was loaded per well and separated on SDS-PAGE followed by transfer to a PVDF membrane. The membrane was blocked with 5% (wt/vol) skimmed milk overnight and probed with rabbit-anti-mouse polyclonal anti-p-mTOR antibody (S2448, 1 : 1000, Abcam, USA) at 4°C overnight and then rinsed with TBST three times. The membrane was then incubated with HRP-conjugated goat anti-rabbit IgG (1 : 10000, Jackson ImmunoResearch Inc.) at room temperature for 1.5 h followed by TBST rinsing three times. Enhanced chemiluminescence (ECL) reagents were added (Thermo Fisher Scientific, USA) and the membrane was exposed in the dark. Protein quantitative analysis was conducted by Image-Pro Plus 6.0 after scanning the film. The results were expressed as optical density and the ratio of the optical density of the p-mTOR protein band to corresponding GAPDH protein in each group was statistically analyzed.

### 2.8. Statistical Analysis

Data with normal distribution and homogeneity of variance were analyzed using Student's *t*-test. Other data sets were analyzed by Mann-Whitney *U* test. Results were presented as mean ± SEM, and *P* < 0.05 was considered significant.

## 3. Results

### 3.1. Effect of the Chinese Herbal Mixture on the Body Weight Gain of Rats

**Figure 1:**
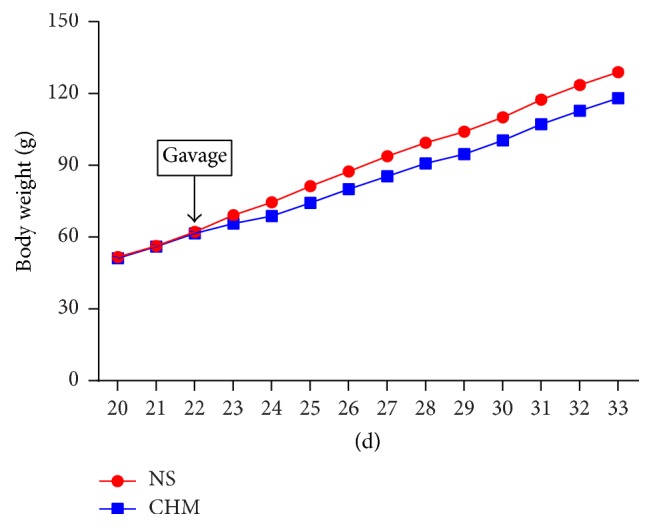
Effect of the Chinese herbal mixture on the body weight gain of rats. In the first 3 days (d22, d23, and d24) after beginning gavage, rats in CHM group showed a decrease in everyday body weight gain comparing with those in NS group (*P* < 0.05). In the following days from d25, there was no significant difference in everyday body weight gain between the two groups (*P* > 0.05). NS: normal saline, CHM: Chinese herbal mixture.

The changes of body weight of 34-day-old rats were observed in two groups (*n* = 10, Figure 1). Before gavage, there was no significant difference in body weight between rats in the two groups. In the first 3 days (d22, d23, and d24) after beginning gavage, rats in CHM group showed a decrease in everyday body weight gain comparing with those in NS group (*P* < 0.05). The decrease may be due to the adaptation of the Chinese herbal mixture as, in the following days from d25, there was no significant difference in everyday body weight gain between the two groups (*P* > 0.05).

### 3.2. Effects of the Chinese Herbal Mixture on the Time of Vaginal Opening

**Figure 2:**
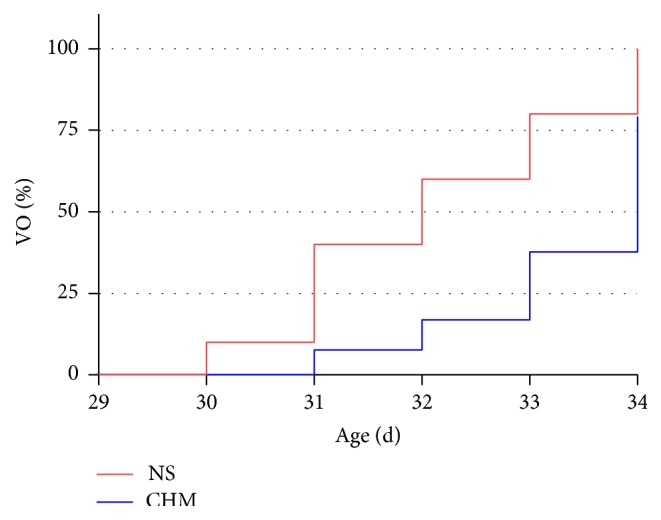
Effect of the Chinese herbal mixture on the vaginal opening time. By d32, vaginas of 60% rats in NS and only 20% in CHM had opened. By d34, vaginas of 100% rats in NS and 80% in CHM had opened. The average VO time in CHM was significantly delayed compared to that in NS. NS: normal saline, CHM: Chinese herbal mixture.

The vaginal opening (VO) time of 34-day-old rats was observed in two groups (*n* = 10, Figure 2). By d34, rats in the NS group had all completed VO, while only 8 rats in the CHM group had their vaginas open. The average time of VO was analyzed and the day of VO of rats with unopened vaginas was recorded as 35 days. The mean VO time in CHM (33.5 ± 0.4 d) was significantly delayed in comparison with that in NS (32.1 ± 0.4 d) and the difference was statistically significant (*P* < 0.05).

### 3.3. Effects of the Chinese Herbal Mixture on Wet Weight and Organ Coefficients of Uterus and Ovary

As shown in Table 4, on d31, the wet weight and the organ coefficients of uteri and ovaries in the CHM group were significantly lower than those in the NS group (*P* < 0.05). As shown in Tables 3 and 5, on d28 and d34, the wet weight and organ coefficients of uteri and ovaries were not significantly different between the two groups (*P* > 0.05), except for the organ coefficients of uteri on d34, which were higher in the CHM group.

### 3.4. Effect of the Chinese Herbal Mixture on Ovarian Morphology

**Figure 3:**
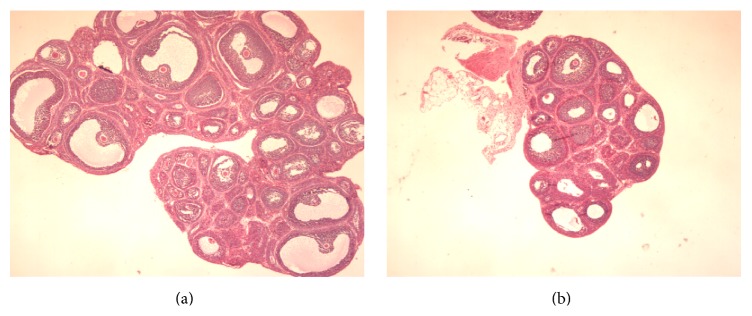
Effect of the Chinese herbal mixture on ovarian morphology (40x). Figure shows the HE-stained paraffin sections of ovaries of rats at d31 in two groups. Ovaries from the NS group had plenty of antral follicles and large follicular cavities (a), and ovaries in the CHM group were less developed and contained small antral follicles and small follicular cavities (b).

The HE-stained paraffin sections of ovaries from animals in both groups were analyzed (Figure 3). At d31, ovaries from the CHM group contained small antral follicles and small follicular cavities; ovaries from the NS group had plenty of antral follicles and large follicular cavities, showing that ovaries in the NS were well developed and mature.

### 3.5. Effects of the Chinese Herbal Mixture on Serum Hormone Levels

**Figure 4:**
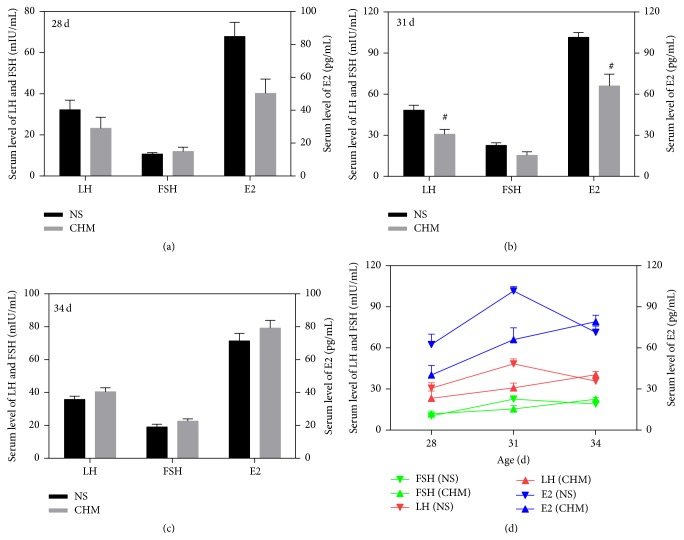
Effects of the Chinese herbal mixture on serum LH, FSH, and E2 levels. At d31, serum LH and FSH levels in CHM were significantly lower (*P* < 0.05) than those in NS. Due to the action of the Chinese herbal mixture on rats in the CHM group, the serum levels of LH, FSH, and E2 slowly increased (d). NS: normal saline, CHM: Chinese herbal mixture. ^#^ *P* < 0.05.

As shown in Figure 4, at d28 (*n* = 5) and d34 (*n* = 10), there was no significant difference in the serum LH, FSH, and E2 levels between the two groups (*P* > 0.05). At d31 (*n* = 5), however, serum hormone (LH and E2) levels in CHM were significantly lower than that in NS (*P* < 0.05). At all the three time points, FSH levels showed no significant differences between two groups (*P* > 0.05); however, at d31, FSH level in CHM seemed to show a downward trend comparing with those in NS (22.67 ± 1.90 mIU/mL versus 15.44 ± 2.53 mIU/mL).

Figure 4(d) shows the changes in rat serum hormone levels during puberty onset. At d28, rats in CHM and NS were both at an early stage of puberty; therefore, the serum LH, FSH, and E2 levels were low. At d31, rats in NS were on their way to puberty, and the serum LH, FSH, and E2 levels sharply increased to peak levels, but in CHM, they increased slowly. At d34, serum LH, FSH, and E2 levels in NS gradually declined, but in CHM, the serum hormone levels continued increasing slowly to the peak levels.

### 3.6. Effects of the Chinese Herbal Mixture on mTOR mRNA and p-mTOR Protein Expression in the Hypothalamus

**Figure 5:**
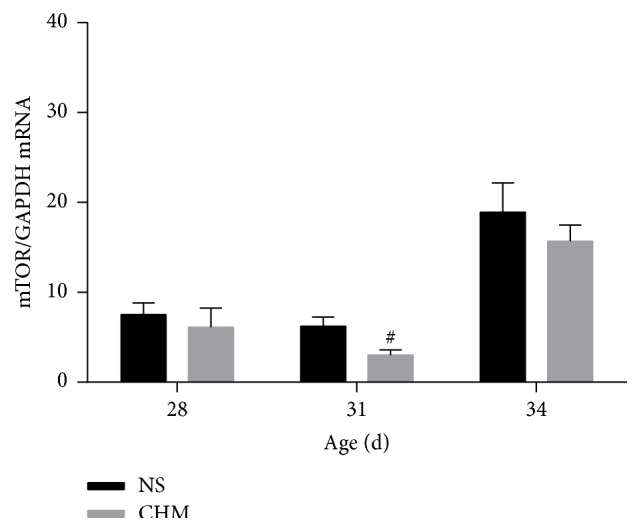
Effects of the Chinese herbal mixture on mTOR mRNA in hypothalamus. During the onset of puberty, mTOR mRNA expression levels in CHM were significantly lower at d31 (*P* < 0.05). At d28 and d34, there was no significant difference in the expression levels of mTOR mRNA between the two groups. NS: normal saline, CHM: Chinese herbal mixture. ^#^ *P* < 0.05.

**Figure 6:**
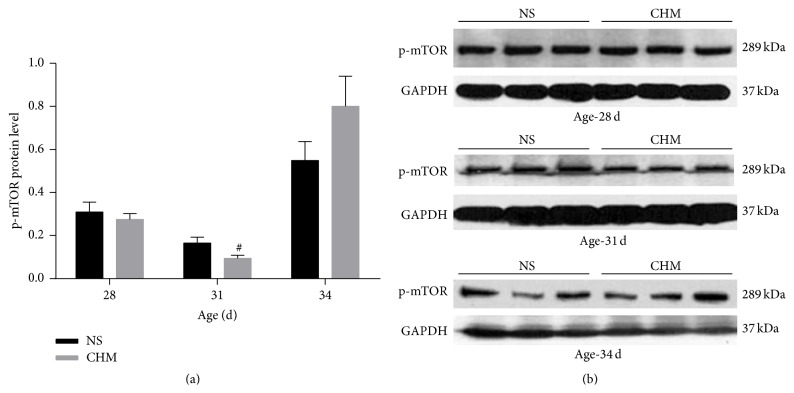
Effects of the Chinese herbal mixture on p-mTOR expression in the hypothalamus. During the onset of puberty, p-mTOR protein expression levels in CHM were significantly lower at d31 (*P* < 0.05). At d28 and d34, there was no significant difference in the expression levels of p-mTOR between the two groups. NS: normal saline, CHM: Chinese herbal mixture. ^#^ *P* < 0.05.

At d28 (*n* = 5) and d34 (*n* = 10), there was no significant difference in hypothalamic mTOR mRNA expression between the two groups (*P* > 0.05). At d31 (*n* = 5), hypothalamic mTOR mRNA expression in CHM was significantly lower than that in NS (*P* < 0.05) (Figure 5). At d28 (*n* = 3) and d34 (*n* = 3), there was no significant difference in hypothalamic p-mTOR protein expression levels between the two groups (*P* > 0.05). At d31 (*n* = 3), hypothalamic p-mTOR protein expression level of rats in CHM was significantly lower than that in NS (*P* < 0.05), which was consistent with the mRNA changes (Figure 6).

## 4. Discussion

A number of large-scale retrospective epidemiological studies suggest that girls in developed countries tend to experience early puberty, which is characterized by breast development at Tanner II stage (sign of puberty onset of girls) and earlier average age at menarche [9, 20, 21]. In China, there is a lack of national large-scale epidemiological studies, but some local investigations report that there is a tendency of early menarche age [22, 23]. Some recent studies have found that early age at menarche may be associated with high risk of metabolic diseases such as diabetes, cardiovascular disease, breast cancer, and even asthma [24–26], and this condition has attracted wide social attention. Chinese herbal mixtures can significantly delay the onset of puberty and age at menarche, slow down the process of early epiphyseal closure in children with early puberty/precocious puberty, and improve their final adult height. Therefore, it is meaningful to study the mechanism by which Chinese herbal mixtures affect puberty onset. Our study is the first to explore the effects of Chinese herbal mixture on the hypothalamus mTOR signaling, aiming to elucidate the mechanism by which this Chinese herbal mixture delays puberty onset.

As the body weight* per se* had an impact on the puberty onset, the effect of Chinese herbal mixture on the body weight gain was investigated in our study. We found that, only in the first three days of gavage, the body weight gain showed a decrease in CHM which may be due to the bitterness of the herbs; from the fourth day on, the body weight gain of rats in the two groups showed no significant differences which suggested that the Chinese herbal mixture only had a mild effect on the body weight. In fact, a mild decrease in body weight had little effect on puberty development [8].

In our study, we found that the vaginal opening in rats exposed to CHM was significantly delayed, and at d31, the wet weight of ovaries and uteri and the coefficients of uteri and ovaries in the CHM group were significantly lower than those in the NS group. The histology of paraffin sections of ovaries indicated that the ovary development in rats from the CHM group was slower than that in the NS group, suggesting that the Chinese herbal mixture can significantly delay the puberty onset in female rats.

Due to the dramatic changes in hormone levels during the process of puberty, different time points were chosen (d28, d31, and d34) in our study to analyze the serum hormone levels during puberty development. These results showed that, only at d31, rats in CHM group had significant differences in the puberty relevant indicators as compared to the NS group. At d34, there was no significant difference between the two groups, indicating that the effects of the herbal mixture on puberty were in timeliness. At d28, rats in the CHM group showed decreased serum LH and E2 levels after administration of the herbal mixture for a week. However, rats at this time were still at an early stage of puberty and the hormone levels themselves were low. Therefore, the herbal mixture may have small inhibitory effects on the hormone levels that were not statistically significant. At d31, rats in the NS group gradually started puberty, so hormone levels of LH, FSH, and E2 quickly increased to the peak, but in CHM, hormone levels slowly increased due to suppression by herbal mixture on the HPGA. At this time, the inhibition of herbal mixture on serum hormone levels reached significance. At d34, hormone levels in NS began to decline after the peak at d31, but in CHM, it was seen that the peaks in serum hormone levels were delayed due to the HPGA suppression by herbal mixture. From Figure 4(d) we can see that serum hormone levels in CHM increased gradually and reached their peaks. When rats in CHM group were at d34, the hormones showed higher levels than that in the NS group. At this time, the inhibitory effect of the Chinese herbal mixture on HPGA was largely antagonized by high hormone levels, and uteri and ovaries of rats in CHM rapidly matured by the action of hormones. Our findings suggested that this Chinese herbal mixture functioned slowly but effectively and its inhibitory effects on HPGA were obvious especially in the short period just before puberty onset.

mTOR is a principal regulatory protein, and Roa et al. [8] found that the inhibition of hypothalamic mTOR signaling can delay the onset of puberty in female rats, which was consistent with our experimental data (unpublished). In our study, RT-PCR results showed that hypothalamic mTOR mRNA expression on d31 in CHM group was significantly lower than that in the NS group, indicating that this Chinese herbal mixture can inhibit the mTOR gene transcription. The western blot results showed that hypothalamic p-mTOR protein expression level on d31 in the CHM group was significantly lower than that in the NS group. The serum hormone levels were not fully in accordance with the hypothalamic mTOR mRNA and protein expression, which is likely due to the complexity of neuroendocrine regulatory networks. Our research suggests that this Chinese herbal mixture can reduce the hypothalamic mTOR gene expression and protein levels, preliminarily inferring that the mechanism by which the Chinese herbal mixture delays puberty onset is partially related to inhibition of the mTOR signaling.

## 5. Conclusion

Nourishing “Yin” removing “Fire” Chinese herbal mixture can delay the onset of puberty in female rats and downregulate the expression of mTOR mRNA and p-mTOR protein. The mechanism of this effect is likely related to the hypothalamic mTOR signaling.

## Figures and Tables

**Table 1:** Composition of nourishing “Yin” removing “Fire” herbal mixture.

Chinese name	Botanical name	Family	Common name	Used part
Sheng-Di-Huang	*Rehmannia glutinosa*	Scrophulariaceae	Rehmannia root	Dried root tuber
Xuan-Sheng	*Scrophularia buergeriana*	Scrophulariaceae	Buerger's Figwort	Dried root tuber
Zhi-Mu	*Anemarrhena asphodeloides*	Liliaceae	Zhimu	Dried rhizome
Huang-Bai	Cortex Phellodendri	Rutaceae	Phellodendron bark	Dried bark
Mu-Dan-Pi	*Paeonia suffruticosa* Andr.	Ranunculaceae	Moutan	Bark dried root
Xia-Ku-Cao	*Prunella vulgaris* L.	Lamiaceae	Common self-healing	Dried aerial parts and flowers
Gui-Jia	Carapax et Plastrum Testudinis	Testudinidae	Plastron of fresh-water tortoise	Carapace and plastron of the turtle *Chinemys reevesii*
Long-Dan-Cao	*Gentiana scabra* Bge.	Gentianaceae	Chinese gentian	Dried root and rhizome

**Table 2:** Primer sequences.

Name of gene	Primer sequence (5′ to 3′)	Amplification fraction
GAPDH	Forward: ACTTTGGCATCGTGGAAGGG	128 bp
	Reverse: TGCAGGGATGATGTTCTGGG	

mTOR	Forward: CAATTATACTCGCTCCCTGGCT	131 bp
	Reverse: AGCAGTCCCCAAAGTCAATGT	

**Table 3:** Wet weights and organ coefficients of uteri and ovaries at d28 (*n* = 5).

Groups	Wet weight of uteri (g)	Organ coefficients of uteri	Wet weight of ovaries (g)	Organ coefficients of ovaries
NS	0.0721 ± 0.0049	8.11 ± 0.41	0.0208 ± 0.0013	2.36 ± 0.19
CHM	0.0580 ± 0.0058	7.01 ± 0.82	0.0175 ± 0.0009	2.10 ± 0.12

There were no significant differences between the two groups. NS: normal saline, CHM: Chinese herbal mixture.

**Table 4:** Wet weights and organ coefficients of uteri and ovaries at d31 (*n* = 5).

Groups	Wet weight of uteri (g)	Organ coefficients of uteri	Wet weight of ovaries (g)	Organ coefficients of ovaries
NS	0.1699 ± 0.0241	15.89 ± 2.04	0.0451 ± 0.0049	4.29 ± 0.55
CHM	0.0646 ± 0.0196^#^	6.73 ± 1.99^#^	0.0232 ± 0.0021^#^	2.42 ± 0.19^#^

The wet weight and organ coefficients of uteri and ovaries in CHM were significantly lower, which suggested that the Chinese herbal mixture could significantly delay the development of uteri and ovaries of pubertal female rats. NS: normal saline, CHM: Chinese herbal mixture. ^#^ *P* < 0.05 versus NS.

**Table 5:** Wet weights and organ coefficients of uteri and ovaries at d34 (*n* = 10).

Groups	Wet weight of uteri (g)	Organ coefficients of uteri	Wet weight of ovaries (g)	Organ coefficients of ovaries
NS	0.1366 ± 0.0162	10.97 ± 1.33	0.0434 ± 0.0035	3.46 ± 0.25
CHM	0.1513 ± 0.0162	13.57 ± 1.48^#^	0.0366 ± 0.0023	3.28 ± 0.20

NS: normal saline, CHM: Chinese herbal mixture. ^#^ *P* < 0.05 versus NS.
